# Molecular detection and antibiogram of *Staphylococcus aureus* in rabbits, rabbit handlers, and rabbitry in Terengganu, Malaysia

**DOI:** 10.5455/javar.2021.h527

**Published:** 2021-09-19

**Authors:** Min Hian Chai, Muhammad Zikree Sukiman, Nurlailasari Mohammad Najib, Nor Arifah Mohabbar, Nur Aina Nadhirah Mohd Azizan, Noor Muzamil Mohamad, Siti Mariam Zainal Ariffin, Mohd Faizal Ghazali

**Affiliations:** 1 School of Animal Science, Aquatic Science and Environment, Faculty of Bioresources and Food Industry, Universiti Sultan Zainal Abidin, Besut, Terengganu, Malaysia; 2 Centralised Laboratory Management Centre, Universiti Sultan Zainal Abidin, Besut, Terengganu, Malaysia; 3 Department of Veterinary Preclinical Sciences, Faculty of Veterinary Medicine, Universiti Putra Malaysia, Serdang, Malaysia

**Keywords:** Rabbit, *Staphylococcus aureus*, MRSA, *nuc* gene, *mecA* gene, antibiogram

## Abstract

**Objectives:**

This study aims to investigate the prevalence and antibiogram of *Staphylococcus aureus* and methicillin-resistance *S. aureus* (MRSA) in rabbits, rabbit handlers, and rabbitry environments in Terengganu.

**Materials and Methods:**

Swab samples from 183 rabbits (183 oral and 183 ear swabs), 45 rabbit handlers (45 oral and 45 nasal), and environmental (*n* = 180) samples from rabbitries were collected from 10 rabbit farms in Terengganu. The associated *S. aureus* isolates from the swabs were isolated using phenotypic microbiology tests. The bacteria were confirmed by polymerase chain reaction targeting *nuc* (*S. aureus*) and *mecA* (MRSA) genes. The antibiogram of all *S. aureus* isolates was determined using the Kirby–Bauer test.

**Results:**

*Staphylococcus aureus* was detected in 19% of rabbits, 26.7% of rabbit handlers, and 8.8% of swabs from the rabbitry environment. However, MRSA (0%) could not be detected. Antibiotic susceptibility test revealed that *S. aureus* from rabbits showed low resistance (<20%) against 15 different antibiotics while fully susceptible to 4 antibiotics. Meanwhile, *S. aureus* from rabbit handlers showed high resistance against penicillin (86%), oxacillin (64%), and amoxicillin (50%).

**Conclusions:**

This study suggests the emergence of antibiotic-resistant *S. aureus* in rabbit farms settings. Therefore, careful selection of antimicrobial agents will be essential to preserve the effectiveness of treatments toward *S. aureus* infections.

## Introduction

*Staphylococcus aureus* is a zoonotic, opportunistic Gram-positive, commensal bacterium colonizing humans and animals [1]. *Staphylococcus aureus* is capable of causing several diseases in humans, ranging from minor skin infections to life-threatening illnesses [1]. In rabbits, *S. aureus* can be commonly found on the skin. It is one of the primary pathogens related to dermatitis, mastitis, and metritis infections, causing losses in the rabbit farming industry [2,3]. A previous study indicated that both asymptomatic carriers and infected individuals could transmit *S. aureus* to others via direct and indirect pathways [4].

Recently, the emergence of bacteria with antimicrobial resistance (AMR) traits such as *S. aureus* is a significant public health concern in human and veterinary medicines. Methicillin-resistant *S. aureus* (MRSA) is one of the critical widespread nosocomial pathogens seen worldwide. The occurrence of MRSA in animals has been reported worldwide. The apparent animal-to-human transmissions have raised concerns on the risks of animal populations as potential reservoirs for this zoonotic infection [5]. In addition, there has been increasing attention to the possible roles of the environment as a potential reservoir of MRSA. Previous studies reported MRSA detection from various environmental samples associated with animals such as dust, farm rats, and environmental wipes [6]. 

Similar to other animals, rabbits are also prone to various bacterial infections [3]. Medication level in rabbit farming is the highest among food-producing animals [7]. Thus, this creates an optimum environment for the emergence of AMR bacterium. Recently, rabbits carrying *S. aureus* with AMR traits have been reported [3]. Moreover, livestock-associated MRSA ST398 has been described to be present in both farm and pet rabbits [2,8]. In Malaysia, although most of the rabbit farms are considered to be small scale, they still have the potential risk for the spread of AMR. Thus, it is advisable to implement surveillance plans to regulate antibiotic usage and prevent the emergence of multidrug-resistance bacteria. However, no study is documented regarding the prevalence and antibiogram of *S. aureus* and MRSA from rabbit farms in Malaysia. Hence, this study investigates the prevalence and determines the antibiogram of *S. aureus* and MRSA isolated from rabbits, rabbit handlers, and farm environments in Terengganu, Malaysia. 

## Materials and Methods

### Ethics approval

The sampling and experimental design method of this study were approved by UniSZA Animal and Plant Research Ethics Committee (Protocol code: UAPREC/04/18/006.) and UniSZA Human Research Ethics Committee (Protocol code: UniSZA/UHREC/2019/85). Written informed consent was obtained from each rabbit handler before obtaining swabs samples.

### Swabs samples collection

Swab samples (183 oral and 183 ear swabs) from 183 randomly selected rabbits of various ages from 10 different rabbit farms in Terengganu were taken using sterilized cotton swabs. Ear swab samples were collected by swabbing both the external ear canals of the rabbit. Oral swab sample was collected by swapping the back of throat and tonsil of the rabbits for a few seconds. Besides, 90 swab samples (45 oral and 45 nasal swabs) were also collected from 45 human handlers having close contact with rabbits. The oral swabs were taken by swapping the back of the throat, while nasal samples were collected by swabbing the anterior nares of the animal handlers. For environmental samples, a total of 180 swabs were taken from feeders (*n* = 30), drinkers (*n* = 30), door lock (*n* = 30), cage wall (*n* = 30), and floor (*n* = 30) of randomly selected rabbits’ cages as well from the footwears of the rabbit handlers (*n* = 30). The swab samples were kept in modified transport media containing nutrient broth (HiMedia, India) supplemented with 6.5% sodium chloride (NaCl) and stored at 4°C. The samples were transported from the sampling sites to the laboratory within 24 h for analysis.

### Bacteriological examination

The samples were inoculated onto mannitol salt agar plates (Sigma-Aldrich, USA) before being incubated for 48 h at 37°C. The appearances of the bacteria colonies were monitored and recorded at 24 and 48 h. Bacterial colonies with the yellow round appearance (yellow colored as a result of mannitol fermentation) similar to *S. aureus* were picked, and sub-cultured on the nutrient agar (NA) plates (HiMedia, India) was supplemented with 6.5% of sodium chloride (NaCl). The NA plates then underwent an incubation process at 37°C for 24 h. The growth of bacteria was then evaluated using Gram stain and biochemical tests (catalase test, oxidase test, and coagulase test). Bacteria isolates showing phenotypic characteristics similar to *S. aureus* were kept under 4°C before polymerase chain reaction (PCR) testing. 

### Genotypic identification of bacterial isolates

The genomic DNA of the isolated bacteria was extracted via the heat lysis method described by Suhaili et al. [1]. The DNA templates were stored at −20°C before genotypic identification analysis. DNA amplification of *nuc* (detection of *S. aureus*) and *mecA* (detection of MRSA) genes were then carried out using PCR [9]. PCR products were loaded into 2.0% (w/v) agarose gel (Promega, USA), and gel-electrophoresis was run at 80 V for 2 h. The gels were visualized and documented using the Fujifilm LAS-4000 gel documentation system. Bacteria that showed DNA bands at 278 (*nuc* gene) and 533 base pair (bp) (*mecA* gene) were considered MRSA. Bacterial isolates that only harbored *nuc* genes were referred to as methicillin-susceptible *S. aureus* (MSSA).

### Antibiotic susceptibility test

*Staphylococcus aureus* isolated from rabbits and rabbit handlers were subjected to Kirby–Bauer test to determine their antibiogram profile [10]. The turbidity of bacterial suspensions was adjusted to 0.5 McFarland standards [11]. The Kirby–Bauer test was carried out following the guidelines set by the Clinical and Laboratory Standards Institute (CLSI) [11]. Nineteen different antibiotic disks were placed on Mueller Hinton agar plates inoculated with 0.5 McFarland standardized *S. aureus* cultures. The antibiotic disks used in this study include penicillin (10 units), oxacillin (1 μg), amoxicillin/clavulanate (10 μg), tetracycline (30 μg), erythromycin (15 μg), clindamycin (2 μg), cefotaxime (30 μg), trimethoprim–sulfamethoxazole (25 μg), amikacin (30 μg), cefoxitin (30 μg), chloramphenicol (30 μg), cephalothin (30 μg), ciprofloxacin (5 μg), doxycycline (30 μg), gentamicin (30 μg), kanamycin (30 μg), linezolid (30 μg), norfloxacin (10 μg), and quinupristin-dalfopristin (15 μg). The plates were then incubated at 37°C for 24 h. The inhibition zones of each antibiotic were measured and interpreted according to the disc diffusion breakpoints given by CLSI [11]. *Staphylococcus aureus* isolates that showed resistance to three and more classes of antibiotics were categorized as multidrug-resistance *S. aureus* isolates [12]. 

### Data analysis

The number of *S. aureus* and MRSA were counted and presented in percentages. The number of *S. aureus* that showed resistance against all antibiotics was calculated and presented as antibiotic resistance rate (%). Categorical data were compared and analyzed using the Pearson Chi-square test or Fisher’s exact test (Minitab^®^ 16.1.1, 2010), with a 95% confidence interval (*p* < 0.05) was set to indicate the significant difference. The prevalence of antibiotic resistance was presented as the proportion of isolates tested with an inhibition zone (diameter) below the respective antibiotic breakpoint. The relationships between antibiotic exposure and overall antibiotic resistance in *S. aureus* isolates were assessed using a multiple antimicrobial resistance index (MARI). The MARI was calculated as the proportion of antibiotics tested to which the isolate was phenotypically resistant. A dendrogram based on the phenotypic antibiotic resistance profile was generated using unweighted pair group method with arithmetic mean method (UPGMA) in BioNumerics 8.0 software (Applied Maths, TX) to visualize the relatedness of the *S. aureus* isolated from rabbit and rabbit handlers. 

## Results and Discussions

**Figure 1.**
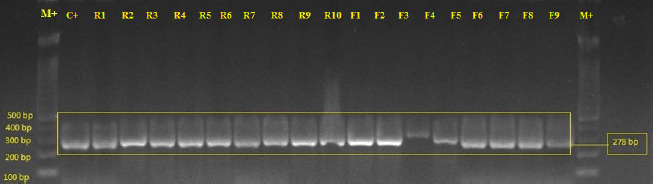
Agarose gel electrophoresis image of *nuc* gene (278 bp) from representative *S. aureus* isolates. R represents rabbits and F represents rabbit handlers. Lane labelled M+ is the DNA ladder and lane labelled C+ is the control (ATCC700699).

Post-screening of the *nuc* gene (Fig. 1) had resulted in a total of 40 *S. aureus* samples isolated from 35 different rabbits (19.1%; 35/183) as mentioned in Table 1. A total of 18 (45%; 18/40) *S. aureus* isolates were from the ear while the remaining 22 (55%; 22/40) isolates came from the oral swabs of rabbits. The prevalence result was lower than previous studies that reported 41%–63% of *S. aureus* occurrence rate in lesion swab samples [13,14]. Genotypic analysis revealed that 26.7% (12/45) of animal handlers carried *S. aureus*, where most isolates were oral. This finding agrees with the statement given by Kozajda et al. [15], where 20%–40% of the human population were *S. aureus* carriers. However, Agnoletti et al. [2] reported *S. aureus* among 58.3% of individuals related to rabbit farms. The differences in *S. aureus* prevalence rates in both rabbits and handlers may be due to geographical factors, different sampling sites, and sampling size. 

In this study, only 8.8% (16/180) of the environmental swabs were *S. aureus*-positive, indicating a low *S. aureus* contamination rate at the surface of feeder, drinker, door lock, wall and floor of cages, and feet wear of farmers and their families (Table 2). Further data analysis revealed that *S. aureus* was mainly found on drinkers (16.7%; 5/30), suggesting that drinkers and drinking water may play a role in the transmission and dispersion of *S. aureus* among the rabbits. *Staphylococcus aureus* isolated from the drinker surfaces may be originated from the rabbits or from the water itself. However, as the water sources from the farms were not tested, the possibility of *S. aureus* originated from the drinking water remained unclear. Apart from drinkers, *S. aureus* was also found on boots or slippers (13.3%), door lock (10%), wall of the cage (6.7%), the floor of the cage (3.3%), and feeder (3.3%). Previous studies also reported *S. aureus* on the surface of various farm objects, suggesting that various environmental surfaces in rabbit farms may act as a vehicle of transmission for *S. aureus* [2,6,16].

**Table 1.** Prevalence rate of *S. aureus* and MRSA based on *nuc* and *mecA* gene detection.

No.	Samples	No. of individual carrying *S. aureus* (%)	No. of individual carrying MRSA (%)
1.	Rabbits	35 (19.1%)	0 (0%)
2.	Rabbit handlers	12 (26.7%)	0 (0%)
3.	Environment	16 (8.9%)	0 (0%)

**Table 2.** Occurrence rate of *S. aureus* and MRSA according to sampling site in the rabbit farm environment.

No.	Sampling sites	No. of samples	No. of samples positive for *nuc* gene	No. of samples positive for *mecA* gene
1.	Feeder	30	1	0
2.	Drinker	30	5	0
3.	Door lock	30	3	0
4.	Wall of cage	30	2	0
5.	Floor of cage	30	1	0
6.	Boots/slippers	30	4	0

In the present study, all *S. aureus* samples isolated from rabbit handlers, rabbits, and environments were categorized as MSSA as they did not carry any *mecA* gene. Briefly, *mecA* is a chromosomally derived gene responsible for producing penicillin binding protein, which helps MRSA develop resistance against antibiotics from the beta-lactam class. The *mecA* gene is common in MRSA but absent in methicillin-susceptible *Staphylococci* isolates. Today, many have considered detecting the *mecA* gene using PCR as the most reliable and “gold standard” technique to identify MRSA [17]. In this study, none of the *S. aureus* harbored the *mecA* gene, indicating the absence of MRSA. The finding agrees with the study of Attili et al. [3], which reported a 0% MRSA prevalence rate among live rabbits from Italy. However, another previous study reported MRSA among rabbit handlers and rabbits, with the prevalence rate recorded at 32% and 3%, respectively [2]. Furthermore, the presence of MRSA in environments such as veterinary hospitals and rabbit holdings was also reported [2].

The emergence of AMR in important pathogens of humans and animals is a significant concern. In the present study, *S. aureus* from rabbit and rabbit handlers showed different resistances against 18 selected antibiotics. However, the antibiogram of *S. aureus* from humans (Table 3) and animals (Table 4) differed. In the rabbit, *S. aureus* showed a relatively low level of resistance (below 20%) against 15 antibiotics. All the rabbit farm owners claimed that no antibiotics were used during the rearing process, which might explain the low antibiotic resistance rate displayed by *S. aureus*. A similar result was also reported by Wang et al. [14], where the antibiotic-resistance rates of *S. aureus* against penicillin (11%), kanamycin (19.6%), gentamicin (10%), and ciprofloxacin (3.9%) were less than 20%. However, a previous study conducted in Italy reported that most of the *S. aureus* isolated from rabbits were highly resistant against tetracyclines (96%) and macrolides (94%) [3].

Nonetheless, *S. aureus* from rabbit showed the highest resistance rate against chloramphenicol (15%; 6/40). The presence of chloramphenicol resistant *S. aureus* in rabbits is surprising as chloramphenicol usage in the livestock industry is banned [18]. However, there is a possibility that the rabbits were exposed to chloramphenicol through the consumption of plants that have been contaminated with naturally occurring chloramphenicol produced by *Streptomyces venezuelae* in the soil [19]. However, as no samples were taken from the feed and the soil, the possible source of chloramphenicol resistance in *S. aureus* from rabbits remained uncertain. 

Meanwhile, *S. aureus* isolated from rabbit handlers showed resistance against five different antibiotics, including penicillin (86%; 12/14), oxacillin (64%; 9/14), amoxicillin/clavulanate (50%; 7/14), tetracycline (14%; 2/14), and erythromycin (7%; 1/14). This finding was not surprising as -lactam antibiotics such as penicillin are often prescribed to treat bacterial infections in humans. According to Che Hamzah et al. [20], 84.4% of MSSA isolated from Hospital Sultanah Nur Zahirah in Malaysia were resistant to penicillin. However, it is noteworthy that the oxacillin resistance rate displayed by *S. aureus* from rabbit handlers in this study was higher than the 5.5% resistance prevalence rate given by the previous study [20]. Thus, the usage of -lactam antibiotics to treat *S. aureus* infections in humans may no longer be a viable choice shortly. Nonetheless, all *S. aureus* isolated from rabbits and their handlers were fully susceptible to cephalothin, cefoxitin, and kanamycin, suggesting that these antibiotics can be used to treat persistent *S. aureus* treatment for both rabbit and rabbit handlers in the selected rabbit farms. 

**Figure 2.**
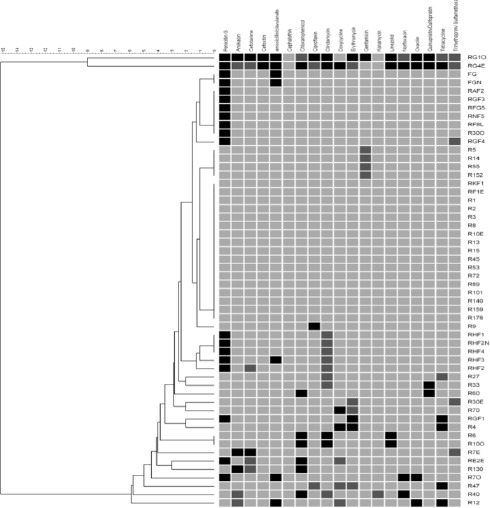
Dendrogram illustrating the relatedness of *S. aureus* based on their phenotypic antibiotic resistance pattern. Black colour columns = resistance, dark gray columns = intermediate resistance, and light gray columns = susceptible.

Three of the *S. aureus* isolates (one from humans and two from rabbits) were found to be multidrug-resistant *S. aureus* (MDRSA) [21]. This result is lower than the 93% of MDRSA prevalence rate in rabbits reported elsewhere [3]. Another study by Silva et al. [22] revealed that 16 MRSA isolated from pus samples of rabbits were all multidrug-resistant. Nonetheless, the presence of MDRSA in rabbits should be a concern as AMR-carrying bacterium may spread further to other animals and humans. The emergence of AMR bacteria strain was often the result of intensive and unregulated use of antimicrobial drugs in human and veterinary medicine [10,23]. Thus, it is possible that these MDRSA from both humans and rabbits came from an environment with high antibiotic usage. MARI assessment was carried out to assess this possibility. The data (Table 5) showed that two of *S. aureus* from rabbits had MARI values of 0.2 and above, indicating the isolates originated from environments with frequent antibiotic usage. In addition, a dendrogram (Fig. 2) was generated based on the antibiogram profile of the *S. aureus* showed high diversity among the isolates, indicating that this *S. aureus* may have significant differences in antibiotic exposure genetic background. 

**Table 3.** Antibiogram of *S. aureus* isolates from rabbits (*n* = 40).

No.	Antibiotics	Number of isolates (%)		
		Susceptible	Intermediate	Resistant
1.	Chloramphenicol	34 (85%)	0 (0%)	6 (15%)
2.	Amoxicillin/clavulanate	36 (90%)	0 (0%)	4 (10%)
3.	Clindamycin	36 (90%)	0 (0%)	4 (10%)
4.	Linezolid	36 (90%)	0 (0%)	4 (10%)
5.	Norfloxacin	36 (90%)	0 (0%)	4 (10%)
6.	Oxacillin	30 (75%)	6 (15%)	4 (10%)
7.	Penicillin	36 (90%)	0 (0%)	4 (10%)
8.	Quinupristin-Dalfopristin	36 (90%)	0 (0%)	4 (10%)
9.	Tetracycline	36 (90%)	0 (0%)	4 (10%)
10.	Amikacin	36 (90%)	2 (5%)	2 (5%)
11.	Cefotaxime	36 (90%)	2 (5%)	2 (5%)
12.	Ciprofloxacin	36 (90%)	2 (5%)	2 (5%)
13.	Doxycycline	36 (90%)	2 (5%)	2 (5%)
14.	Erythromycin	38 (95%)	0 (0%)	2 (5%)
15.	Gentamicin	35 (87.5%)	4 (10%)	1 (2.5%)
16.	Trimethoprim–Sulfamethoxazole	36 (90%)	4 (10%)	0 (0%)
17.	Cefoxitin	40 (100%)	0 (0%)	0 (0%)
18.	Cephalothin	40 (100%)	0 (0%)	0 (0%)
19.	Kanamycin	40 (100%)	0 (0%)	0 (0%)

**Table 4.** Antibiogram of *S. aureus* isolates from rabbit handlers (*n* = 14).

		Number of isolates (%)		
No.	Antibiotics	Susceptible	Intermediate	Resistant
1.	Penicillin	1 (7.6%)	0 (0%)	13 (92.8%)
2.	Oxacillin	5 (36%)	0 (0%)	9 (64%)
3.	Amoxicillin/clavulanate	3 (21%)	4 (29%)	7 (50%)
4.	Tetracycline	12 (86%)	0 (0%)	2 (14%)
5.	Erythromycin	13 (93%)	0 (0%)	1 (7%)
6.	Clindamycin	10 (71%)	4 (29%)	0 (0%)
7.	Cefotaxime	13 (93%)	1 (7%)	0 (0%)
8.	Trimethoprim–Sulfamethoxazole	13 (93%)	1 (7%)	0 (0%)
9.	Amikacin	14 (100%)	0 (0%)	0 (0%)
10.	Cefoxitin	14 (100%)	0 (0%)	0 (0%)
11.	Chloramphenicol	14 (100%)	0 (0%)	0 (0%)
12.	Cephalothin	14 (100%)	0 (0%)	0 (0%)
13.	Ciprofloxacin	14 (100%)	0 (0%)	0 (0%)
14.	Doxycycline	14 (100%)	0 (0%)	0 (0%)
15.	Gentamicin	14 (100%)	0 (0%)	0 (0%)
16.	Kanamycin	14 (100%)	0 (0%)	0 (0%)
17.	Linezolid	14 (100%)	0 (0%)	0 (0%)
18.	Norfloxacin	14 (100%)	0 (0%)	0 (0%)
19.	Quinupristin-Dalfopristin	14 (100%)	0 (0%)	0 (0%)

**Table 5.** MARI assessment of *S. aureus* isolates from rabbits and handlers (*n =* 54).

Resistance to number of antibiotics	Number of isolates	Percentages (%)	MARI
0	32	59.3	0
1	13	24.1	0.05
2	6	11.1	0.11
3	1	1.9	0.16
4	0	0	0.21
5 and above	2	3.7	0.26

## Conclusion

This study showed that the *S. aureus* had been successfully isolated from rabbit, rabbit handlers, and the environment. No MRSA was identified, but the presence of MDRSA was detected in both rabbits and rabbit handlers. *Staphylococcus aureus* from rabbit handlers showed resistance against penicillin, oxacillin, amoxicillin/clavulanate, tetracycline, and erythromycin. Meanwhile, *S. aureus* from rabbits showed resistance against 15 different antibiotics. These findings suggest the emergence of antibiotic-resistant *S. aureus* that is present in the rabbit farms settings. Therefore, antibiotic usage in rabbits needed to be regulated to prevent the further emergence of AMR. Antimicrobial stewardship programs need to be conducted to educate the rabbit farm owners and workers regarding the risk of AMR. More surveillance programs investigating the AMR in rabbits from other states of Malaysia should be conducted to better understand the AMR issues in the Malaysian rabbit farming industry.

## List of Abbreviations

°C, degree Celsius; %, percentage; μg, microgram; AMR, antimicrobial resistance; bp, base pair; MDRSA, multidrug-resistant *Staphylococcus aureus*; MARI, multiple antibiotic resistance index; MRSA, methicillin-resistant *Staphylococcus aureus*; MSSA, methicillin susceptible *Staphylococcus aureus*; NaCl, sodium chloride; *S. aureus*, *Staphylococcus aureus.*

## References

[1] Suhaili Z, Rafee PA, Mat Azis N, Yeo CC, Nordin SA, Abdul Rahim AR (2018). Characterization of resistance to selected antibiotics and panton-valentine leukocidin-positive *Staphylococcus aureus* in a healthy student population at a Malaysian university. Germs.

[2] Agnoletti F, Mazzolini E, Bacchin C, Bano L, Berto G, Rigoli R (2014). First reporting of methicillin-resistant *Staphylococcus aureus* (MRSA) ST398 in an industrial rabbit holding and in farm-related people. Vet Microbiol.

[3] Attili AR, Bellato A, Robino P, Galosi L, Papeschi C (2020). Analysis of the antibiotic resistance profiles in methicillin-sensitive *S. aureus* pathotypes isolated on a commercial rabbit farm in Italy. Antibiotics.

[4] Smith TC, Gebreyes WA, Abley MJ, Harper AL, Forshey BM, Male MJ (2013). Methicillin-resistant *Staphylococcus aureus* in pigs and farm workers on conventional and antibiotic-free swine farms in the USA. PLoS One.

[5] Smith TC (2015). Livestock-associated *Staphylococcus aureus*: the United States experience. PLoS Pathog.

[6] Friese A, Schulz J, Zimmermann K, Tenhagen BA, Fetsch A, Hartung J (2013). Occurrence of livestock-associated methicillin-resistant *Staphylococcus aureus* in turkey and broiler barns and contamination of air and soil surfaces in their vicinity. Appl Environ Microbiol.

[7] Agnoletti F, Brunetta R, Bano L, Drigo I, Mazzolini E (2018). Longitudinal study on antimicrobial consumption and resistance in rabbit farming. Int J Antimicrob Agents.

[8] Loncaric I, Brunthaler R, Spergser J (2013). Suspected goat-to-human transmission of methicillin-resistant *Staphylococcus aureus* sequence type 398. J Clin Microbiol.

[9] Saiful AJ, Mastura M, Zarizal S, Mazurah MI, Shuhaimi M, Ali AM (2006). Detection of methicillin-resistant *Staphylococcus aureus* using *mecA*/*nuc* genes and antibiotic susceptibility profile of Malaysian clinical isolates. World J Microbiol Biotechnol.

[10] Ariffin SM, Hasmadi N, Syawari NM, Sukiman MZ, Ariffin MFT, Chai MH (2019). Prevalence and antibiotic susceptibility pattern of *Staphylococcus aureus*, *Streptococcus agalactiae* and *Escherichia coli* in dairy goats with clinical and subclinical mastitis. J Anim Health Prod.

[11] CLSI (2018). Performance standards for antimicrobial susceptibility resting. CLSI Supplement M100. 28th edition, Clinical and Laboratory Standards Institute.

[12] Jaja IF, Jaja CI, Chigor NV, Anyanwu MU, Maduabuchi EK, Oguttu JW (2020). Antimicrobial resistance phenotype of *Staphylococcus aureus* and *Escherichia coli* isolates obtained from meat in the formal and informal sectors in South Africa. BioMed Res Int.

[13] Moreno-Grua E, Perez-Fuentes S, Munoz-Silvestre A, Viana D, Fernandez-Ros AB, Sanz-Tejero C (2018). Characterization of livestock-associated methicillin-resistant *Staphylococcus aureus* isolates obtained from commercial rabbitries located in the Iberian Peninsula. Front Microbiol.

[14] Wang J, Sang L, Sun S, Chen Y, Chen D, Xie X (2019). Characterisation of *Staphylococcus aureus* isolated from rabbits in Fujian, China. Epidemiol Infect.

[15] Kozajda A, Jezak K, Kapsa A (2019). Airborne *Staphylococcus aureus* in different environments—a review. Environ Sci Pollut Res.

[16] Friese A, Schulz J, Hoehle L, Fetsch A, Tenhagen BA, Hartung J (2012). Occurrence of MRSA in air and housing environment of pig barns. Vet Microbiol.

[17] Chai MH, Faiq TAM, Ariffin SMZ, Suhaili Z, Sukiman MZ, Ghazali MF (2020). Prevalence of methicillin resistant *Staphylococcus aureus* in raw goat milks from selected farms in Terengganu, Malaysia. Trop Anim Sci J.

[18] EFSA Panel on Contaminants in the Food Chain (CONTAM) (2014). Scientific opinion on chloramphenicol in food and feed. EFSA J.

[19] Berendsen B, Stolker L, De Jong J, Nielen M, Tserendorj E, Sodnomdarjaa R (2010). Evidence of natural occurrence of the banned antibiotic chloramphenicol in herbs and grass. Anal Bioanal Chem.

[20] Che Hamzah AM, Yeo CC, Puah SM, Chua KH, Chew CH (2019). *Staphylococcus aureus* infections in Malaysia: a review of antimicrobial resistance and characteristics of the clinical isolates, 1990–2017. Antibiotics.

[21] Magiorakos AP, Srinivasan A, Carey RB, Carmeli Y, Falagas ME, Giske CG (2012). Multidrug-resistant, extensively drug-resistant and pandrug-resistant bacteria: an international expert proposal for interim standard definitions for acquired resistance. Clin Microbiol Infect.

[22] Silva V, De Sousa T, Gomez P, Sabenca C, Vieira-Pinto M, Capita R (2020). Livestock-associated methicillin-resistant *Staphylococcus aureus* (MRSA) in purulent subcutaneous lesions of farm rabbits. Foods.

[23] Ariffin MF, Hasmadi N, Chai MH, Ghazali MF, Ariffin SMZ (2020). Prevalence and antimicrobial sensitivity pattern of *Staphylococcus aureus* isolated from clinical and subclinical mastitis in small ruminant in Besut and Setiu, Terengganu, Malaysia. Malays J Microbiol.

